# Limb-salvage surgery offers better five-year survival rate than amputation in patients with limb osteosarcoma treated with neoadjuvant chemotherapy. A systematic review and meta-analysis

**DOI:** 10.1016/j.jbo.2020.100319

**Published:** 2020-09-15

**Authors:** Evgenia Papakonstantinou, Alexandros Stamatopoulos, Dimitrios I Athanasiadis, Efstathios Kenanidis, Michael Potoupnis, Anna-Bettina Haidich, Eleftherios Tsiridis

**Affiliations:** a Pediatric Oncology Department, Hippokration Hospital, Thessaloniki, Greece; b Academic Orthopaedic Department, Papageorgiou General Hospital, Aristotle University Medical School, Thessaloniki, Greece; c Center of Orthopaedics and Regenerative Medicine (C.O.R.E.) – Center of Interdisciplinary Research and Innovation (C.I.R.I.), Aristotle University Thessaloniki, Balkan Center, Thessaloniki, Greece; d Department of Surgery, Indiana University School of Medicine, Indianapolis, IN, USA; e Department of Hygiene, Social-Preventive Medicine & Medical Statistics, Medical School, Aristotle University of Thessaloniki, Thessaloniki, Greece

**Keywords:** Osteosarcoma, Amputation, Limb-salvage surgery, Neoadjuvant chemotherapy, AJCC, American Joint Cancer Committee, ASCO, American Society of Clinical Oncology, CATS, Computed Assisted Tumor Surgery, CCG, Children’s Cancer Group, CI, Confidence Interval, COSS, Cooperative Osteosarcoma Study Group, CT, Computed Tomography, DFS, Disease Free Survival, FNA, Fine Needle Aspiration, LSS, Limb Salvage Surgery, MAP, MTX, Adriamycin, Cisplatin, MRI, Magnetic Resonance Imaging, MSKCC, Memorial Sloan Kattering Cancer Center, MSTS, Musculoskeletal Tumor Society, NCCN, National Comprehensive Cancer Network, NOS, Newcastle–Ottawa scale, NPCR, National Program of Cancer Registries, OR, Odds Ratio, OS, Overall Survival, PET, Positron Emission Tomography, POG, Pediatric Oncology Group, RCT, Randomized Controlled Trials, Rev-Man, Review Manager, SIOP, International Society of Paediatric Oncology, SEER, Surveillance, Epidemiology, and End Results, Tc-MDP, Methylene diphosphonate with technetium-99m, VICC, Vanderbilt-Ingram Cancer Center

## Abstract

**Background:**

Osteosarcoma is the most common primary bone sarcoma. Currently, the main treatment option for high-grade osteosarcomas is neoadjuvant chemotherapy, followed by surgical resection of the lesion and adjuvant chemotherapy. Limb salvage surgery (LSS) and amputation are the main surgical techniques; however, controversy still exists concerning the best surgical method. Our meta-analysis compared the effectiveness of LSS and amputation combined with neoadjuvant chemotherapy in patients with limb osteosarcoma, in terms of 5-year overall survival (OS), 5-year disease-free survival (DFS) and local recurrence rate.

**Methods:**

Following the established methodology of PRISMA guidelines, a literature search was conducted in PubMed, Cochrane, Google Scholar from 1975 until January 2020. Two independent reviewers evaluated the study quality based on the Newcastle-Ottawa scale. Odds ratio and 95% confidence interval of the OS, DFS and local recurrence rate were calculated.

**Results:**

Thirteen studies were finally included with a total of 2884 patients; 1986 patients undergone LSS and 898 amputations. Five-year overall survival was almost 2-fold in patients treated with LSS than those treated with amputation (OR: 1.99; 95% CI: 1.35–2.93; I^2^ = 74%, p < 0.001). No difference was found in 5-year DFS between LSS patients and amputees (OR: 1.24; 95% CI: 0.55–2.79; I^2^ = 67%, p = 0.01). The odds of local recurrence was numerically higher in LSS compared to amputation but not statistically significant (OR: 2.29; 95% CI: 0.95–5.53; I^2^ = 47%, p = 0.05). However, the included studies did not clearly define differences in the stages of patients of the two groups.

**Conclusion:**

Our study demonstrated that in patients with limb osteosarcoma treated with neoadjuvant chemotherapy, LSS is associated with a higher 5-year overall survival and the odds of local recurrence may be increased but these results should be interpreted with caution due to high heterogeneity.

## Highlights

• Osteosarcoma is the most common primary bone sarcoma.
• Neoadjuvant chemotherapy combined with limb salvage surgery (LSS) or amputation are the main strategies in treating limb osteosarcoma.
• LSS is associated with a higher 5-year overall survival.
• Local recurrence was more frequently encountered in patients treated with LSS, however not affecting overall survival.

## 1 Introduction

Osteosarcoma is a primary malignant bone tumor of mesenchymal tissue origin affecting mainly the metaphyses of long bones [1]. Osteosarcoma is the most common primary bone sarcoma but less than 1% of all cancer cases [2]. Approximately 7104 new cases of primary osteosarcomas were recognized in the USA between 1999 and 2008 [3]. The peak incidence of osteosarcoma has bimodal age distribution during early puberty and then between the sixth and seventh decade of life [4]. It is slightly more frequent in males; 80% of the reported cases come from femur, tibia, humerus and, pelvis [5].

The overall survival rate of patients with limb osteosarcoma has improved dramatically during the last decades [6]. The surgical removal of osteosarcomas alone without chemotherapy has been used in the past and was often ineffective, as 80% of cases have already metastasized in the lungs at the time of diagnosis [7]. Pulmonary metastases have a median time of appearance at 10 months, a fact that gives a relatively rapid end point for surgery [8]. On the other hand, chemotherapy alone cannot fully control the clinically detectable disease. Currently, the main treatment option for high-grade osteosarcomas is neoadjuvant chemotherapy, including high-dose of methotrexate, doxorubicin, and cisplatin, followed by surgical resection of the lesion and adjuvant chemotherapy [9]. Low-grade osteosarcomas are usually treated with surgical resection alone [10].

There are two main surgical techniques: limb salvage surgery (LSS) and amputation [11]. Limb salvage techniques aim to widely excise of the tumor at the margins of healthy tissue. If this cannot be achieved, amputation is indicated. The type of surgery is determined based on tumor location and size, extramedullary extension, presence of metastatic disease, initial tumor necrosis, age and skeletal development [9]. LSS combined with chemotherapy is the preferable choice of osteosarcoma’s treatment by the majority of surgeons [9]. However, amputation is still supported as an alternative method providing immediate and aggressive removal of osteosarcoma, especially in patients with a pathologic fracture [12].

Despite the enormous progress that has been made in the management of osteosarcoma, the 5-year overall survival does not exceed 70% to 80% [6]. Controversy still exists concerning the best surgical method, mainly due to the presence of a plethora of factors affecting survival, such as the existence of metastasis at diagnosis, the extent of tumor necrosis, the invasion of vessels and nerves, and the disease-free margins after resection [9]. The aim of this meta-analysis is to compare the effectiveness of LSS and amputation in patients with osteosarcoma of the extremities in terms of 5-year overall survival (OS), 5-year disease-free survival (DFS) rates as well as the local recurrence rate of the disease.

## 2 Materials and methods

### 2.1 Study protocol

The systematic review was conducted according to the PRISMA guidelines (Preferred Reporting Items for Systematic Reviews and Meta-Analysis).

### 2.2 Eligibility criteria

#### 2.2.1 Inclusion criteria

Comparative studies between LSS and amputation in humans with limb osteosarcoma were included in the meta-analysis. Eligible studies were randomized controlled trials (RCTs), two-armed prospective and retrospective studies. The search was narrowed to patients surgically treated from primary limb osteosarcoma combined with neoadjuvant chemotherapy, whereas no restriction in the age of patients was imposed. We included studies reporting data on 5-year survival or/and 5-year disease-free survival or/and local recurrence rate.

#### 2.2.2 Exclusion criteria

We excluded studies reporting data from a) bone tumors other than osteosarcoma or non-human subjects; (b) non-comparative studies between LSS group or amputation groups (c) non-limb osteosarcoma regions; (d) less than 25 patients; (e) patients with secondary amputations performed after LSS or for complications; (f) other than the primary or secondary outcomes; (g) patients managed without neoadjuvant chemotherapy or surgical treatment; (h) non-English language studies; (i) patients without follow-up; (j) studies before 1975; (k) editorials, perspectives, letters to the editors, commends, case reports or case series, narrative or systematic reviews.

### 2.3 Study selection and data extraction

#### 2.3.1 Search strategy

We conducted a systematic search in PubMed/MEDLINE (OVID interface, 1948 onwards), Google Scholar, Cochrane Library, Cochrane Central Register of Controlled Trials, Clinicaltrials.gov and ISRCTN registry for relevant studies published from 1975 to January 2020. Conference and meeting abstracts as well as presentations reported in the American Society of Clinical Oncology and International Society of Paediatric Oncology, were also scanned. We used the following medical subject heading terms “osteosarcoma”, “amputation”, “limb-salvage surgery” and “limb-sparing surgery” in combination with Boolean operators (AND, OR). We used the PRESS (Peer Review of Electronic Search Strategies) checklist to evaluate the quality of our search strategies.

We followed the Cochrane Handbook for Systematic Reviews of Interventions. Two reviewers independently screened potentially eligible articles after reading the title and abstract according to the inclusion criteria. To exclude duplicates, they compared the author’s names, institution, sample size, and baseline characteristics of the patients, along with the date and duration of the study. When additional information and supplemental material was needed, the corresponding author of the paper was contacted. The reference lists of all potentially included articles were also hand-searched. Any disagreement was solved through consensus, and where considered necessary, a third investigator was asked to evaluate the study independently.

#### 2.3.2 Data extraction

Data were extracted from each study by two independent reviewers using a standardized data extraction form. Data extracted from each article were publication year, country, study period, design and type of study, number of participating centres, and patients, Enneking stage, patients’ demographics, follow up period, tumor location, margins, response in neoadjuvant chemotherapy, fractures and metastases at diagnosis and tumor size. Conflicts were resolved through discussion and when necessary, a third author evaluated each study independently to solve the disagreement.

### 2.4 Outcome measures

The primary outcome measurement was the 5-year OS rate. The secondary outcomes included 5-year disease-free survival (DFS) and local recurrence of the disease.

### 2.5 Quality assessment

Two independent reviewers assessed the methodological quality of the eligible studies the modified Newcastle-Ottawa scale (NOS). It includes eight items and uses a “system” where studies can be given up to nine stars. High-quality trials scored six or more points, with a maximum of nine points [13]. NOS is focused on three areas of interest namely the selection, comparability and exposure of study participants: Inclusion criteria, sample size >25, endpoint, anatomical location, Enneking stage, follow up >60 months, 5-year overall survival, 5-year disease-free survival, local relapse. A third reviewer was responsible for the estimation of the discrepancies.

### 2.6 Statistical analysis

This meta-analysis was elaborated with Review Manager (Revman) Software (version 5.4 for Windows. Copenhagen: The Nordic Cochrane Centre, The Cochrane Collaboration, 2020). The statistical heterogeneity between studies was assessed with the Chi-square test. P-values < 0.10 were used to determine statistical significance. The extent of heterogeneity between the trial results was assessed with the I^2^ statistic of inconsistency. I^2^ value ≥ 75% was defined as high heterogeneity, 50–74% moderate and 25–49% low [14]. Moreover, the Z statistic was estimated for the final effect. We calculated pooled odds ratios (ORs) and 95% CI for dichotomous data according to the Mantel-Haenszel method. We used random-effects model to pool the OR, based on the heterogeneity of the studies included in the meta-analysis (different chemotherapy protocols, surgical techniques, surgical margins, tumor necrosis and follow up time).

We used funnel plots of 5-year overall survival, 5-year disease-free survival and local recurrence to identify publication bias; asymmetric plots may suggest publication bias. Then we conducted sensitivity analyses based on study quality to exclude outlier studies. The quality of evidence was evaluated with GRADE (Grading of Recommendations Assessment, Development and Evaluation system).

## 3 Results

### 3.1 Literature search

**Fig. 1:**
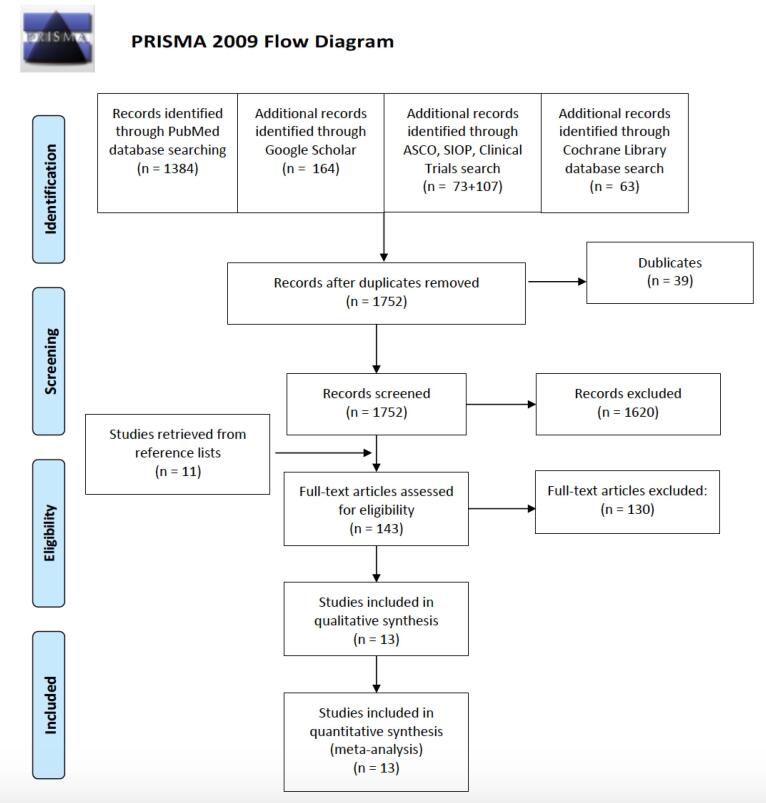
PRISMA (Preferred Reporting Items for Systematic Reviews and Meta-Analysis) guidelines flowchart demonstrating the search strategy.

The primary literature search revealed 1791 (1384 + 164 + 107 + 73 + 63) relevant articles. After removal of duplicates and screening for title and abstracts, we excluded (1620 + 39) articles (Fig. 1). Finally, 143 articles were considered potentially eligible and were studied in full text. One hundred and seven articles were excluded based on the inclusion criteria, while 23 articles were excluded, as they studied the same patient population, estimated from the institution, number of patients and name of the authors. At last, 13 articles met the inclusion criteria and were eligible for analysis [15], [16], [17], [18], [19], [20], [21], [22], [23], [24], [25], [26], [27]. Screening and selection details are depicted in Fig. 1.

### 3.2 Study characteristics

All eligible studies were retrospective. The majority of studies were conducted in a centre, but four of them were multi-institutional. The articles were published between 1992 and 2019. The study period ranged from 1977 to 2015. A total of 2884 patients were studied; 1986 patients underwent LSS and 898 amputations of the involved limb. The sample size of studies ranged from 58 to 560 patients. In the majority of studies, the number of patients was higher for the LSS compared to amputation group. The stage of osteosarcoma ranged from I to III. However, the included studies did not clearly define differences in the stages of patients of the two groups. The mean age of the patients was 24 ± 15 years, and the mean follow-up was 80 ± 27.8 months. The main characteristics of the involved studies are summarized in Table 1 and the demographics and baseline data of patients in Table 2. The main outcomes of the study, as well as data connected with the outcomes, are presented in Table 3, Table 4.

**Table 1:** The main characteristics of the included studies.

Authors	Study period	Study design	Center	OS Stage	Country of origin
Fujiwara et al.	2007–15	R	A	IIA-B	UK
Han et al.	2000–15	R	A	IA-B, IIA-B	China
Faisham et al.	2005–10	R	A	I-III	Malaysia
Zhang et al.	2006–12	R	A	IIA-B	China
Kamal et al.	1995–2014	R	A	IIB, III	Indonesia
Deng et al.	NA	R	M	Ι-ΙΙ	India
Wu et al.	1992–2002	R	A	IIB	China
Schrager et al.	1988–2007	R	M	Ι, II, III	USA
Shih et al.	1991–2000	R	A	IIA-B, III	China
Bacci et al.	1983–1995	R	A	I, IIA-B, III	Italy
Grimer et al.	1983–1993	R	M	I,II	UK, Nederland’s
Sluga et al.	1977–1990	R	A	II,III	Austria
Tsuchiya et al.	1980–85	R	M	IIA-B, III	Japan

OS: osteosarcoma, R: retrospective, A: one center, M: multicentre.

**Table 2:** The baseline characteristics, demographic and clinical data of treatment groups.

Authors	Number of patients	L/A	M-F (L/A)	Age (years)	Follow-up (months)
Fujiwara et al.	226	173/53	136–90	25.3 (19.1)*	61 (6–120)^&^
Han et al.	79	52/27	30–22/12–15	28.8 (7.9)* [L: 25.5 (8.7), A: 31.2 (6.4)]	79 (12–158)^&^95 (15–142)^&^
Faisham et al.	117	79/38	79–38	25.8 (15.8)*	53.6 (14.4)*
Zhang et al.	112	72/40	44–28/20–20	19.4(11–46)^&^ [L: 16.59 (6.51), A: 18.85 (8.01)]	52.2 (22.7)* [L: 53.9 (22.17), A: 52.23 (22.72)]
Kamal et al.	79	37/42	48–31	18.2 (6.8)*	NA
Deng et al.	95	59/36	55–40	16 (8–51)^&^	27 (1–223)^&^
Wu et al.	58	43/15	30–28	27 (13)*	130.8 (34.8)*
Schrager et al.	890	590/300	317–273/179–121	15 (1–19)^&^	60^**^
Shih et al.	86	71/15	48–23/7–8	17 (11–45)^&^	64.8(27.6–115.2)^&^
Bacci et al.	560	465/95	263–202/57–38	23.8(<14), 32.2(14–40)^&^	126 (60–204)^&^
Grimer et al.	202	154/48	133–69	19.8 (10.3)*	109 (20.8)*
Sluga et al.	130	84/46	42–42/27–19	17 (5–21)^&^	97.1 (64.4)*
Tsuchiya et al.	250	107/143	52–55/88–55	28.5 (21,7)*	71.4 (22)*

L/A: limb salvage procedure/amputation, M-F: male-female, NA: non-answered.

*: Values are given as a mean with standard deviation in parentheses.

&: Values are given as a mean with range in parentheses.

**: Values are given as a mean.

**Table 3:** The 5-year survival rate, 5-year disease free survival and local recurrence rate of treatment groups per study.

Authors	Survival(L/A)*	DFS(L/A)*	Local recurrence(L/A)*
Fujiwara et al.	128(74)/33(63)	154(89)/52(98)	18(10.4)/1(1.9)
Han et al.	45(86.5)/21(77.8)	NA	6(11.5)/0(0)
Faisham et al.	46(58)/5(13)	NA	NA
Zhang et al.	35(48.6)/18(45)	NA	12(16.7)/2(5)
Kamal et al.	13(34.8)/7(15.9)	36(96.2)/36(86.5)	1(2.7)/6(14.3)
Deng et al.	39(66)/17(46.8)	NA	5(8.5)/1(2.8)
Wu et al.	NA	19(44.2)/8(53.3)	7(16.3)/1(6.7)
Schrager et al.	429(72.7)/180(60.1)	NA	NA
Shih et al.	35(49.3)/2(13)	35(49.3)/1(6.66)	8(11.3)/1(6.7)
Bacci et al.	230(49.4)/60(63.2)	200(63)/47(49.4)	30(6.4%)/4(4.2%)
Grimer et al.	92(60)/16(33.8)	NA	21(13.6)/0(0%)
Sluga et al.	61(73)/29(64)	60(71)/28(60)	1(1.2)/2(4.3)
Tsuchiya et al.	75(70)/70(49)	NA	15(14)/NA

L/A: limb salvage procedure/amputation, N/A: non-answered.

*: Values are given as raw numbers with percentages in parentheses.

**Table 4:** Additional characteristics of the included studies.

Authors	Bone site	Poor chemotherapy response*	Pathologic fracture*	Metastatic occurrence(L/A) *
Fujiwara et al.	femur, tibia, fibula, humerus	109	27	0/0
Han et al.	tibia	NA	NA	0/0
Faisham et al.	femur, tibia, humerus	L:41, A:0	17	28/16
Zhang et al.	tibia	NA	NA	0/0
Kamal et al.	femur, tibia, fibula, humerus	44	NA	13/18
Deng et al.	femur, tibia, fibula, humerus	ΝΑ	95	0/0
Wu et al.	femur, tibia, fibula	ΝΑ	ΝΑ	0/0
Schrager et al.	NA	NA	NA	85/63
Shih et al.	femur, tibia	NA	NA	0/10
Bacci et al.	femur, tibia, humerus	L:145, A:49	L:50, A:23	0/0
Grimer et al.	femur, tibia, fibula, humerus	L:123, A:47	13	0/0
Sluga et al.	femur, tibia, fibula, humerus	L:29, A:22	16	5/5
Tsuchiya et al.	NA	L:29, A:65	NA	4/17

L: limb salvage procedure, A: amputation, NA: non-answered.

*: Values are given as raw numbers.

### 3.3 Quality assessment/risk of bias in included studies

The methodological quality of studies varied. The median quality score of the involved studies was 7 (range 6–9). All of the included studies were of high quality according to the modified Newcastle-Ottawa scale score. Data on bias assessment are depicted in Table 5.

**Table 5:** Quality assessment for the 14 articles based on Newcastle- Ottawa quality assessment scale.

	Selection			Comparability			Exposure			
Author	1	2	3	4	5	6	7	8	9	NOS
Fujiwara 2019	*	*	*	*	–	*	*	*	–	7*
Han 2017	*	*	*	*	–	*	*	–	–	6*
Faisham 2017	*	*	*	*	*	–	*	–	–	6*
Zhang 2017	*	*	*	*	*	–	*	–	*	7*
Kamal 2016	*	*	*	*	*	–	*	*	*	8*
Deng 2015	*	*	*	*	–	–	*	–	*	6*
Wu 2012	*	*	*	*	*	*	–	*	*	8*
Schrager 2011	*	*	*	*	–	*	*	–	–	6*
Shih 2005	*	*	*	*	*	*	*	*	*	9*
Bacci 2002	*	*	*	*	*	*	*	*	*	9*
Grimer 2002	*	*	*	*	–	*	*	–	*	7*
Sluga 1999	*	*	*	*	*	*	*	*	*	9*
Tsuchiya 1992	*	*	*	*	*	*	*	*	–	8*

1, Inclusion criteria; 2, sample size >50; 3, endpoint; 4, anatomical location; 5, Enneking stage; 6, follow up >60mo; 7, 5-year overall survival; 8, 5-year disease free survival; 9, local relapse; NOS, Newcastle- Ottawa scale score.

### 3.4 Differences in 5-year overall survival.

**Fig. 2:**
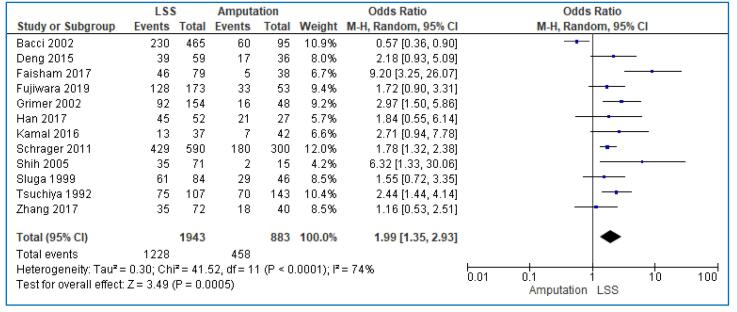
Forest plot comparing the 5-year overall survival rate for Limb Salvage Surgery (LSS) vs Amputation for the treatment of limb osteosarcoma.

We included 12 studies [15], [16], [17], [18], [19], [20], [21], [22], [23], [24], [25], [27] analyzing the 5-year overall survival rate in 2826 patients; 1943 patients were treated with LSS and 883 with amputation. The overall pooled analysis demonstrated that the odds of 5-year overall survival was almost 2-fold in patients treated with LSS than those treated with amputation but with substantial heterogeneity (OR: 1.99; 95% CI: 1.35–2.93; I^2^ = 74%, p < 0.001) (Fig. 2). The funnel plot was symmetric, suggesting that publication bias was unlikely to be present (Fig. 5).

### 3.5 Differences in 5-year DFS

**Fig. 3:**
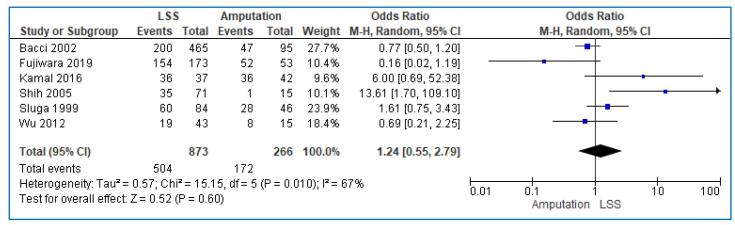
Forest plot comparing the 5-year disease-free survival (DFS) of the patients treated with limb salvage surgery (LSS) vs amputation for the treatment of limb osteosarcoma.

A total of six studies [15], [18], [21], [23], [24], [26], involving 1139 patients evaluated the 5-year DFS of patients with limb osteosarcoma; 873 patients were treated with LSS and 266 with amputation. The pooled analysis showed that the 5-year DFS was not different between those treated with LSS and those treated with amputation (OR: 1.24; 95% CI: 0.55–2.79; I^2^ = 67%, p = 0.01) (Fig. 3). The prevalence of the funnel plot symmetry indicated no evidence of publication bias (Fig. 5).

### 3.6 Differences in local recurrence

**Fig. 4:**
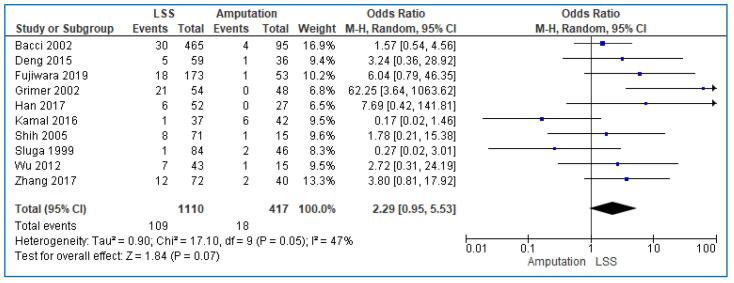
Forest plot comparing the local recurrence rate of the patients treated with limb salvage surgery (LSS) vs amputation for the treatment of limb osteosarcoma.

**Fig. 5:**
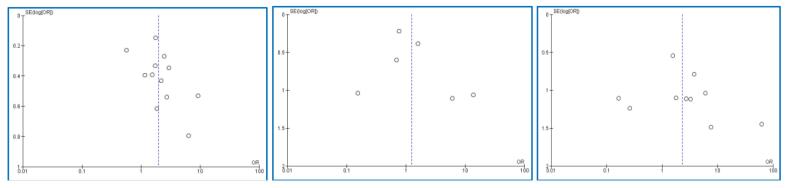
Funnel plot comparing the **a.** 5-year overall survival **b.** 5-year DFS **c.** Local recurrence rates of LSS or amputation.

Ten studies [15], [16], [18], [19], [20], [21], [23], [24], [26], [27] encompassing 1527 patients reported data on the local relapse of the disease; 1110 patients were treated with LSS and 417 with amputation. A pooled analysis of these eleven studies demonstrated the higher local recurrence of patients treated with LSS than those treated with amputation; however, this difference was non-significant (OR: 2.29; 95% CI: 0.95–5.53; I^2^ = 47%, p = 0.05) (Fig. 4). The symmetric funnel plot recommended again that publication bias was non-probable (Fig. 5).

### 3.7 Sensitivity analysis

We performed a sensitivity analysis of the included studies in order to determine the reliability of the results. We performed forest plots of 5-year OS rates, 5-year DFS rates, local recurrence rates between the LSS and amputation groups, with each study removed in turn. The direction and magnitude of the combined estimates did not change markedly with the exclusion of individual studies, indicating that the results of the meta-analysis are reliable.

### 3.8 Subgroup analysis

**Fig. 6:**
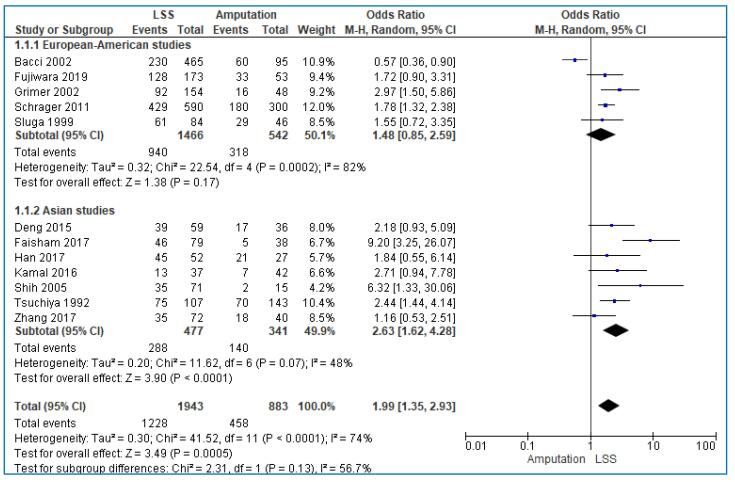
5-year OS in European-American versus Asian studies.

**Fig. 7:**
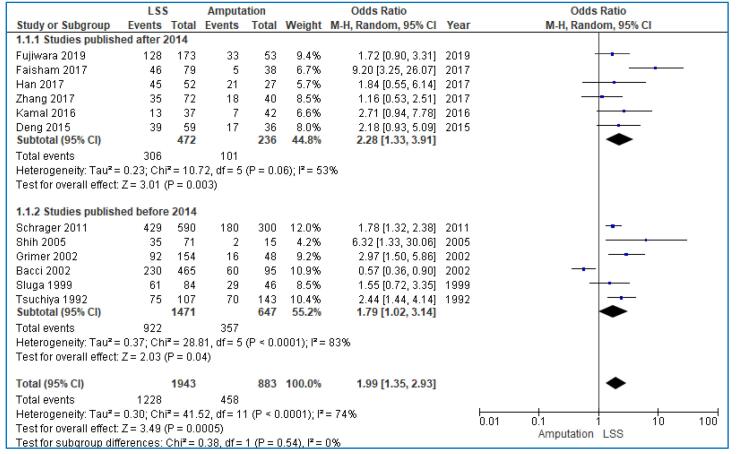
5-year OS in studies published before 2014 versus after 2014.

We also conducted subgroup analysis for 5-year OS, because of the heterogeneity of the included studies. The first subgroup analysis included the European-American studies compared to the Asian studies. The pooled analysis showed that the 5-year OS was not different between those treated with LSS and those treated with amputation in European-American studies (OR: 1.48; 95% CI: 0.85–2.59; I^2^ = 82%, p < 0.001), in contrast with Asian studies in which LSS has significantly better 5-year OS (OR: 2.63; 95% CI: 1.62–4.28; I^2^ = 48%, p = 0.07) (Fig. 6). A second subgroup analysis compared the studies published before 2014 and those published after 2014. As shown in the forest plots, our results were similar in both groups (Fig. 7).

## 4 Discussion

The survival of patients suffering from osteosarcoma has been improved during the last 50 years. Following the successful administration of neoadjuvant chemotherapy and the advances in surgical techniques, a considerable improvement in the overall survival of patients with primary osteosarcoma has been achieved [10]. On the other hand, the effect of the type of surgical treatment on survival has not been estimated yet as different studies yielded conflicting results. Our meta-analysis compared survival and local recurrence in patients with limb osteosarcoma treated with LLS vs amputation. Our study demonstrated that for patients with primary limb osteosarcoma following neoadjuvant therapy, LSS provided significantly higher 5-year survival rate than amputation. However, no difference was found in 5-year DFS between groups. Although the local recurrence rate after LSS treatment was higher than amputation, the difference was not statistically significant too. Unfortunately, the existing literature did not clearly describe the differentiations in stages and prognosis between the two groups, that could affect the comparison between groups.

In the majority of studies of our meta-analysis, the 5-year overall survival was significantly higher in patients treated with LSS than those treated with amputation for patients with osteosarcoma of the limbs. Only the study of Bacci et al. [15] found that the amputation resulted in better overall survival than LSS. Bacci et al. mentioned that limb-salvage procedures are associated with a reduction of surgical margins, a fact that can increase the incidence of local recurrence. In their series, patients who had local recurrence faced poorer outcome with a five-year survival of only 6%. They supported that limb-salvage procedures can lead in inadequate surgical margins, increase of local recurrence and subsequent worse survival. Our results are partially consistent with previous meta-analyses. The meta-analysis that was reported by Han et al. [28] included 11 studies and found a higher 5-year survival rate in the LSS compared with amputation group, but no differences in 2-year survival rate between the groups [28]. Their analysis for 5-year overall survival included eight studies and concluded that patients receiving LSS had significantly better results than those receiving amputation [28]. On the other hand, the meta-analysis from He et al. that included ten studies supported that patients managed either with LSS or amputation had similar 5-year survival [11]. Excluding three studies that caused heterogeneity and whose subjects were Asians; however, they found that the 5-year overall survival rate was higher in patients treated with LSS than those treated with amputation [11]. Previous meta-analyses included studies in which patients did not receive preoperative chemotherapy. In our study, however, we included only patients that received neoadjuvant chemotherapy preoperatively.

A recent systematic review [29] reported similar survival rates between the two procedures, but higher local recurrence rates for LSS as compared to amputation (8.2% versus 3.0%) [29]. Local recurrence is expected to be higher in LSS group as a general rule as the margins in an amputation will usually be radical. These results are consistent with the results of the meta-analyses of Han et al. and Li et al. and He et al. Mavrogenis et al. comparing 23 patients with limb osteosarcoma managed with LSS and 19 with amputation found a higher local recurrence rate in the LSS compared to the amputation group (0 vs 13%) [30]. This study was also excluded from our final analysis as some of its data were included in the study of Bacci et al. [15].

The evolution in therapeutic and diagnostic facilities have probably improved disease-free survival rates in patients with osteosarcoma [9]. However, in our study, we found no significant difference in DFS between those patients treated with LSS or amputation. In one of the studies with the higher sample size, Bacci et al. demonstrated a significantly higher 5-year DFS for patients undergoing LSS versus those receiving amputation (63% vs 49%) [15]. Similarly, Sluga et al. found that 5-year DFS was better in patients treated with LSS than amputation (71% vs 60%) [24]. Neo-adjuvant chemotherapy and various staging of patients probably affected our results. Unfortunately, different stages in patients of the two groups may have affected the outcomes.

Because of the significant heterogeneity recognized in our results, we performed 2 distinctive subgroup analyses depending on the continent and the study year of the included studies. The meta-analysis of He et al. was commensurate with our study and showed no difference in 5-year OS between LSS and amputation group. However, when excluding the three studies that caused heterogeneity and included Asian patients, they found that 5-year OS rate of patients treated with LSS was higher than those treated with amputation (OR: 0.50; 95% CI:0.35–0.72; I^2^ = 2%, p = 0.0001). On the other hand, the pooled analysis in our study showed that the 5-year OS was not different between those treated with LSS and those treated with amputation in European-American studies (OR: 1.44; 95% CI: 0.84–2.48; I^2^ = 78%, p = 0.0004), in contrast with Asian studies in which LSS has significantly better 5-year OS (OR: 2.63; 95% CI:1.62–4.28; I^2^ = 48%, p = 0.07). Therefore, it seems that racial calcification can lead to heterogeneity with different results between the two meta-analyses.

Our meta-analysis has several advantages. First of all, in comparison with previous meta-analyses, we included the most recent studies of patients treated with modern surgical techniques. Besides, it is the first study in which a homogenous group of patients that were treated with neoadjuvant chemotherapy was studied. As a result, our meta-analysis does not suffer from selection bias of chemotherapy treatment that was seen in the previous meta-analyses. Moreover, the number of patients included in the meta-analysis was satisfactory, encompassing a total of 2884 patients, with 1986 patients in the LSS and 898 in the amputation arm.

On the other hand, our study has several limitations. First, all studies included were retrospective with a small number of patients. In addition, the criteria of surgical treatment choice were not clarified; possibly, the patients that received amputation were fundamentally those suffering from huge tumors, with poorer response to neoadjuvant chemotherapy and an overall poorer prognosis compared with those who received LSS.

Third, we composed data of patients with different chemotherapy schedules, surgical margins, response to chemotherapy, and initial metastatic disease.

## 5 Conclusions

In conclusion, this meta-analysis represents an essential update to the current literature regarding survival and local recurrence in patients with limb osteosarcoma treated with neoadjuvant chemotherapy and LSS or amputation. Our results showed that LSS resulted in a higher 5-year OS rate compared with amputation. Local recurrence was more frequently encountered in patients treated with LSS, however not affecting overall survival. Although our study provides the most current and comprehensive evidence on the subject, there should be designed comparative studies between patients suffering from osteosarcomas of the same skeletal site and stage to confirm the conclusions.

## Funding

This research did not receive any specific grant from funding agencies in the public, commercial, or not-for-profit sectors.

## Declaration of Competing Interest

The authors declare that they have no known competing financial interests or personal relationships that could have appeared to influence the work reported in this paper.
